# Dosimetric Advantage of Scanning Beam Proton Therapy in Gynecologic Patients Receiving Adjuvant Radiotherapy

**DOI:** 10.3390/cancers17122010

**Published:** 2025-06-17

**Authors:** Rachel B. Ger, Jarrod M. Lentz, Joshua S. Niedzielski, Sujay A. Vora, Martin Bues, Danairis Hernandez Morales, Justin D. Anderson, Christopher J. Kutyreff, Christie A. Schulz, Pedro R. Lara, Ana K. Ridgway, Pamela R. Lemish, Justin D. Gagneur, Aman Anand

**Affiliations:** 1 Department of Radiation Oncology and Molecular Radiation Sciences, Johns Hopkins University School of Medicine, Baltimore, MD 21287, USA; 2 Department of Radiation Oncology, Cancer Care Northwest, Spokane Valley, WA 99216, USA; 3 Department of Radiation Physics, MD Anderson Cancer Center, Houston, TX 77030, USA; 4 Department of Radiation Oncology, Mayo Clinic, Phoenix, AZ 85054, USA; 5 West County Radiological Group, St. Louis, MO 63141, USA; 6 Department of Radiation Oncology, Baptist Health, Louisville, KY 40207, USA; 7 Department of Radiation Oncology, St. Jude Children’s Hospital, Memphis, TN 38105, USA

**Keywords:** gynecological malignancy, proton therapy, NTCP

## Abstract

**Background/Objectives:** Adjuvant radiation for gynecologic malignancies often exposes organs at risk (OARs), such as the bone marrow, bowel, rectum, and bladder, to radiation, leading to toxicities that impact treatment tolerance and patient quality of life. Scanning proton beam therapy, particularly with Individual Field Simultaneous Optimization (IFSO), may offer dosimetric and biological advantages over volumetric modulated arc therapy (VMAT). This study evaluates the clinical impact of IFSO-based proton planning in post-operative gynecologic cancer patients. **Materials and Methods:** Fourteen patients receiving adjuvant proton therapy to 45 Gy in 25 fractions were retrospectively analyzed. Comparison VMAT plans were generated on the same datasets. Dose–volume metrics for key OARs and normal tissue complication probabilities (NTCPs) were compared using paired statistical tests. Robustness evaluations accounted for setup and range uncertainties. **Results:** Proton plans significantly reduced dose to bone marrow (V10Gy: 58% vs. 86%, *p* < 0.00001; V20Gy: 47% vs. 58%, *p* < 0.00001), small bowel (V20Gy: 21% vs. 56%, *p* < 0.00001), and femoral heads (left femoral head mean: 11Gy vs. 13Gy, *p* = 0.032; right femoral head mean: 11Gy vs. 13Gy, *p* = 0.022). NTCP modeling predicted significantly lower rates of bowel urgency (9.4% vs. 3.3%, *p* < 0.001) and hematologic toxicity (10.2% vs. 4.9%, *p* < 0.001) with proton therapy. Plans remained robust across uncertainty scenarios. **Conclusions:** IFSO-based scanning proton therapy provides clinically meaningful sparing of bone marrow and bowel, with the potential to reduce hematologic and gastrointestinal toxicities. These findings support its use in patients receiving adjuvant pelvic radiotherapy, particularly those undergoing extended field treatment or chemotherapy.

## Simple Summary

Pelvic radiation therapy is commonly used after surgery for gynecologic cancers such as endometrial and cervical cancer. However, it can expose nearby healthy organs like the bone marrow, bowel, and bladder to radiation, increasing the risk of side effects such as fatigue, gastrointestinal discomfort, and treatment delays. Proton therapy can more precisely target cancer areas while reducing radiation to the surrounding healthy tissues. In this study, we evaluated a proton therapy planning method called Individual Field Simultaneous Optimization (IFSO) in patients who received post-operative radiation. We compared these proton plans to standard photon-based volumetric modulated arc therapy (VMAT) radiation plans and found that proton therapy significantly reduced radiation to the bone marrow and small bowel—two organs closely linked to important side effects. Additionally, we used biological modeling to estimate the risk of complications and found lower predicted rates of blood count suppression and bowel symptoms with proton therapy. These findings support the potential benefit of IFSO proton therapy, particularly for patients at higher risk of toxicity due to chemotherapy or extended radiation fields.

## 1. Introduction

Gynecological cancer is the second most common cancer worldwide for women, with 1,085,900 estimated new cases annually, primarily cervical, uterine, and ovarian cases [1]. Post-operative radiation is commonly used to treat intermediate- to high-risk endometrial and cervical cancer patients. The radiation fields cover the vagina and draining lymphatics, depending on the extent of the disease. With nearly 35 to 40 percent of patients’ bone marrow present around the treatment volume, it is important to reduce exposure of these organs at risk (OARs) to low doses to mitigate hematological toxicities after treatment [2,3,4] in addition to the rectal and bladder toxicities that can occur due to their close proximity.

Proton beam radiotherapy has been proposed as a treatment modality to reduce the dose to bone marrow and other OARs, with associated low GU and GI toxicity rates [5,6,7,8,9,10,11]. Several studies show a reduction in V10Gy and V20Gy to bone marrow [12,13,14], as well as reduced low-dose volumes of bladder, rectum, and bowel in pelvic malignancies [15,16,17,18,19].

While there is now increased interest in considering pencil beam scanning proton radiotherapy (sPBT) for gynecological malignancies [15,20,21], there remains a paucity of data on advanced treatment planning techniques and their clinical implications. In this study, we aimed to evaluate the dosimetric and biological advantages of sPBT over volumetric modulated arc therapy (VMAT) using a novel technique called Individual Field Simultaneous Optimization (IFSO) [22]. This approach subdivides the target into beam-specific regions, allowing for precise dose conformity and selective organ-at-risk (OAR) sparing, particularly in anatomically complex pelvic geometries.

This study is, to our knowledge, the first to apply IFSO in the adjuvant treatment of gynecologic cancers and to pair this planning approach with both robustness evaluations and normal tissue complication probability (NTCP) modeling. In addition to focusing on frequently cited OARs such as the rectum and bladder, we emphasize the sparing of the bone marrow and small bowel—critical structures in the setting of chemotherapy and extended field radiotherapy. By incorporating both physical and biological modeling and by including para-aortic cases within a post-operative cohort, this study offers additional insight into the potential advantages of modern proton therapy planning techniques for gynecologic malignancies. These preliminary findings may help inform future investigations and contribute to ongoing discussions regarding the clinical implementation of advanced proton planning methods such as IFSO.

## 2. Materials and Methods

This study was performed with a waiver of informed consent from the Mayo Clinic Institutional Review Board. Fourteen post-operational endometrial cancer patients treated with proton therapy in the clinic were selected for inclusion. The same dataset was also used to generate comparison VMAT plans. Demographic and staging information about these patients is summarized in Table 1.

All patients were simulated in the supine position with an empty bladder and immobilized using a Vac-Lok bag (Civco Medical Solutions, Coralville, IA, USA). In addition, at least two fiducial markers were implanted in the apex of the vagina for use as surrogates during the image guidance for their treatments. Pelvis masks (Pelvicast, Orfit Industries, Wijnegem, Belgium) were utilized to move the pannus out of the fields and better stabilize the abdominal region for treatments. In addition, endo-rectal balloons were used in all patients to mitigate excursions of the vaginal target [23]. All scans were performed at a 2.5 mm slice resolution on a Siemens Somatom Definition AS CT scanner (Siemens Healthineers, Erlangen, Germany). All plans considered in this study were taken to a total dose of 45 Gy in 25 fractions. One patient had a sequential boost; the base plan was included, while the boost was excluded from this study. Two patients were treated to 50.4 Gy in 28 fractions, but their plans were scaled to the same dose scheme as the other patients for inclusion in this study. Eight patients received adjuvant chemotherapy after surgery but before radiation therapy. One patient received concurrent chemotherapy and radiation therapy. Two patients’ para-aortic lymph nodes were also treated with radiation. All proton plans were delivered on the Hitachi (Tokyo, Japan) Synchrotron-based beam delivery system, BDS.

### 2.1. Proton Treatment Planning

**Figure 1:**
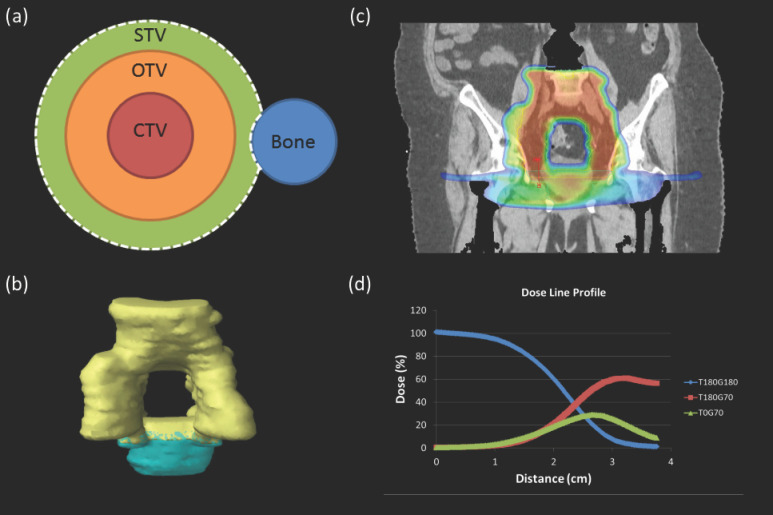
A visual guide to the targets and dose used for gynecological proton planning in our clinic. (**a**) A simple pictorial to demonstrate the relationship of the clinical target volume (CTV), optimization target volume (OTV), and scanning target volume (STV), as well as the cropping of the STV from bone. (**b**) A representative patient’s nodal STV (yellow) and vaginal STV (blue) are shown. The 5–7 mm overlap of these regions is shown where blue patches appear on the yellow. (**c**) A coronal slice of a representative patient is shown with the dose as a color wash, the nodal scanning target volume (STV) in yellow, and the vaginal STV in blue. The red line demonstrates where the dose line profile was taken. (**d**) A plot displaying the dose contributed by each beam along the line profile.

The IFSO method requires generating beam-specific scanning target volumes, which may not cover the entire clinical target volume (CTV), hence creating split targets that cover only a portion of the CTV. This allows for the placement of individual beams that can be strategically arranged to avoid going through heterogeneities. The primary site of disease (vagina and parametrial cavity) and associated nodal chains were divided into sub-targets, which were then used to establish their individual optimization target volume (OTV) and scanning target volume (STV). The OTV represents the equivalent of the planning target volume (PTV) used in photon plans. The STV is the area in which spots can be placed to adequately cover the OTV. Margins for the expansion of these planning structures were governed by the clinical setup margins, in addition to the range uncertainty margins determined by the path lengths of the beam required for each individual case’s treatment planning. On average, a 5 mm OTV for the iliac nodes and a 10 mm OTV for the parametrial/vaginal targets were utilized for our planning. An additional 5 mm of STV for the nodes and 7 mm STV for the parametrial/vaginal targets were used in order to achieve adequate coverage with robustness. The expansion of STVs allowed appropriate coverage, especially in the lateral penumbral regions. It also provided an opportunity to create plans with smooth dose gradients along the junction of the neighboring STVs. Since these expansions of STVs happen to be quite large, it was necessary for almost every case to carefully and selectively crop these contours from various OARs, such as the bladder, the rectum, the bony anatomy, and the bone marrow. A depiction of this method can be seen in Figure 1.

Typically, we used a combination of posterior and anterior oblique beams. The most common arrangement of beam angles was as follows: a posterior beam to treat nodal chains (para-aortic when involved and ilioinguinal lymph nodes), matched with a 5–7 mm overlap with slight anterior oblique beams to treat the vaginal targets. The targets and a dose profile through the target are shown in Figure 1.

IFSO-based optimization is multi-field optimization. A lexicographic ordering of hierarchical optimization was used in the iterative optimization process. Optimization was completed with either Proton Convolution Superposition or a Non-Linear Universal Proton Optimizer, with a 5 mm spot size using either a rectangular or hexagonal grid arrangement. Robust optimization was used as needed, depending on the complexity of the disease and the prescription constraints. Treatment plans were calculated using the Eclipse Treatment Planning System (Varian Medical Systems^®^, Palo Alto, CA, USA) version 13.715.

### 2.2. Photon Treatment Planning

For generating comparative photon plans, we utilized the same CT dataset as was used to plan and treat these patients with sPBT. While gynecological cancer patients treated with photons are often treated with a full bladder, we utilized the empty bladder scan for this study to allow direct comparisons between the proton and photon plans.

The planning structures consisted of planning target volumes, which accounted only for setup-related uncertainties and did not include extra margins to account for range uncertainties. Consequently, the overall planning margins for the photon plans were smaller than those of the proton plans. A PTV was created using a 5 mm uniform expansion of the CTV, which is the current practice for gynecological cancer patients treated with photons in our clinic.

VMAT photon plans were created using four arcs. Two arcs were designed with a collimator angle of 0°, and two arcs were designed with a collimator angle of 90°. The transverse field size for all arcs was limited to 15 cm due to MLC travel limits, which necessitated the use of two arcs at each collimator angle due to the size of the target (Table 1 contains volumetric information on CTVs for patients). The CTV drawn for the clinically treated proton plan was utilized. Plans were created in Eclipse, with dose calculation computed with the Analytical Anisotropic Algorithm version 15.1.51.

### 2.3. Plan Comparisons and Robustness Evaluations

To allow for objective comparison between the proton and photon plans, all plans were normalized to 100% of the prescription dose to cover 99% of the CTV. Dose volume histogram (DVH) metrics were extracted for the bladder, bone marrow, femoral heads, rectum, and small bowel, as shown in Table 2, based on previous proton studies [4,5,7,8,9,10] in gynecological cancer. Specifically, for the rectum, most dose–volume parameters significantly associated with late rectal toxicity are >45 Gy [24]. However, given that the target dose was only 45 Gy in this study, additional lower metrics were included. For bone marrow, V10Gy, V20Gy, V30Gy, and V40Gy were shown to be significantly correlated with hematological toxicity [25]. For the bowel, V45Gy and lower doses are associated with acute toxicities, and RTOG 1203 utilized the V40Gy metric for a planning constraint and demonstrated better bowel function in IMRT plans, which were better able to meet this metric than conventional RT [26,27].

One-sided paired student’s *t*-tests were used to determine if there was a significant (*p* < 0.05) dosimetric improvement in the proton plan compared to the photon plan. As bone marrow is a highly important OAR in this treatment region and was targeted for the dose reduction, it was further explored by comparing the V10Gy dose between the proton and photon plans per patient, as this was a primary endpoint of this study.

In order to ensure the robustness of both proton and photon plans, simulated robustness evaluations were generated within Eclipse, modeling setup uncertainties of 5 mm to ensure plans continued to adequately cover the target and spare the OARs. In addition to the setup uncertainties, range uncertainties of ±3% were also evaluated for the proton plans. Therefore, two separate robustness evaluations were computed on our proton plans. While clinically proton plans would always be evaluated with both setup and range uncertainty, we also evaluated protons with only setup uncertainty to allow for comparison to photon plans with equivalent uncertainty. For each DVH metric, the highest value under each different scenario was selected.

Proton plans were considered robust if 95% of the CTV remained covered with 95% of the prescription dose under all robustness scenarios. Dose gradients along the junction of neighboring STVs were assessed by drawing dose profiles and ensuring the dose falls off from each contributing beam to remain between 2.5 and 3.5% per mm, as can be seen in Figure 1. For all photon plans, the plan was designed to ensure that at least 95% of the PTV was covered by 100% of the prescription dose (prior to normalization for the purpose of this study).

### 2.4. NTCP Evaluations

We used the Lyman–Kutcher–Burman (LKB) normal tissue complication probability (NTCP)-based models published in other works to estimate the complication probabilities for nonuniformly irradiated tissues. These methods are meant to be an in silico surrogate for estimating risks of various toxicities associated with the OARs being studied. In particular, bladder NTCP was assessed using the model by Feng et al. [28] to determine the likelihood of contracture of the bladder neck. Bowel-based urgency syndrome was estimated using the NTCP model from Gulliford et al. [29]. CTCAE Grade 3 or higher hematological toxicity due to bone marrow doses was analyzed through the model by Yoshimura et al. [30], and rectal toxicity involving urgency syndrome was assessed using the NTCP model from Alevronta et al. [31]. Wilcoxon signed-rank tests were performed to determine if there was a significant difference in NTCP between the proton and photon plans.

## 3. Results

The DVH metrics are shown in Table 2. Based on the *t*-tests, the bladder was the only organ that did not show improvement in the proton plan for the extracted metrics. For bone marrow, V10Gy and V20Gy were significantly improved by the proton plan (*p* = 4.5 × 10^−10^ and *p* = 8.3 × 10^−6^, respectively). Mean doses to both femoral heads were significantly improved by the proton plan (left femoral head *p* = 0.03, right femoral head *p* = 0.02). For the small bowel, V20Gy, V30Gy, and V35Gy were all significantly improved in the proton plan (*p* = 3.7 × 10^−8^, *p* = 3.6 × 10^−5^, and *p* = 0.04, respectively). Lastly, the rectum mean dose was improved by the proton plan (*p* = 0.007).

**Table 2:** Dose volume histogram metrics for organs at risk.

Organ	Metric	Photon Mean (Range)	Proton Mean (Range)	*p*-Value
Bladder	Mean	28.6 Gy (23.4–37.6 Gy)	27.6 Gy (20.3–37.2 Gy)	0.17
	V20Gy	67.9% (45.8–84.7%)	64.0% (47.3–86.7%)	0.15
	V45Gy	14.0% (7.6–27.8%)	19.9% (2.1–47.9%)	0.97
Bone Marrow	V10Gy	85.9% (76.3–92.2%)	57.9% (44.5–66.7%)	4.5 × 10^−10^
	V20Gy	58.2% (41.9–67.0%)	47.4% (35.7–59.2%)	8.3 × 10^−6^
	V30Gy	25.2% (16.9–36.6%)	36.1% (29.8–44.1%)	1.0
	V40Gy	10.2% (5.9–17.5%)	14.0% (7.8–21.1%)	1.0
Femoral Head (left)	Mean	12.9 Gy (8.2–18.2 Gy)	11.2 Gy (4.7–16.9 Gy)	0.032
Femoral Head (right)	Mean	12.7 Gy (8.6–18.8 Gy)	10.9 Gy (4.7–16.3 Gy)	0.022
Rectum	Mean	22.9 Gy (15.2–26.8 Gy)	19.7 Gy (9.0–28.1 Gy)	7.4 × 10^−3^
	V30Gy	31.3% (10.4–57.1%)	36.1% (12.6–54.9%)	0.88
	V45Gy	8.0% (0.1–18.1%)	12.9% (1.0–31.4%)	0.98
Small Bowel	V20Gy	56.0% (30.6–74.8%)	21.3% (6.6–53.2%)	3.73 × 10^−8^
	V30Gy	24.5% (8.4–41.4%)	16.9% (4.2–45.5%)	3.59 × 10^−5^
	V35Gy	16.9% (5.0–31.4%)	14.7% (3.2–41.1%)	0.037
	V40Gy	11.8% (3.1−22.7%)	12.1% (2.3–35.8%)	0.59
	V45Gy	6.6% (1.4–14.8%)	7.5% (0.5–26.6%)	0.77

**Figure 2:**
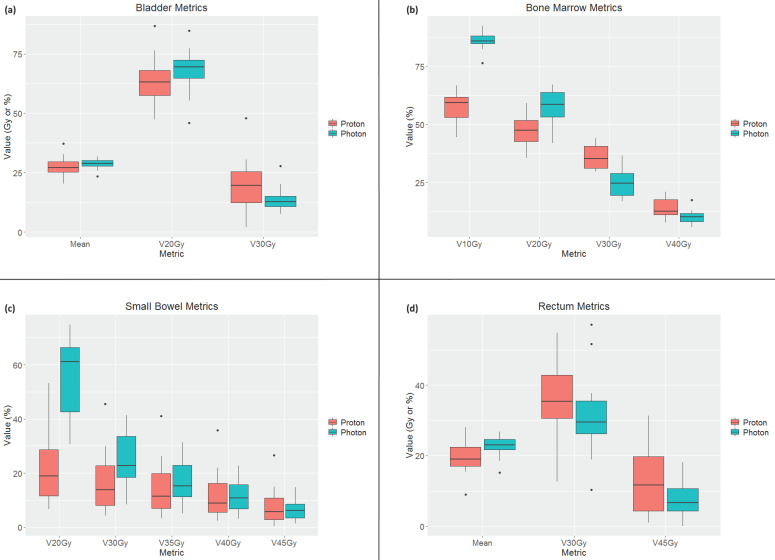
Box plots for the proton plans in red on the left and photon plans in blue on the right for the DVH metrics extracted for (**a**) bladder, (**b**) bone marrow, (**c**) small bowel, and (**d**) rectum. A black dot represents each outlier, defined as a point more than 1.5 times the interquartile range.

**Figure 3:**
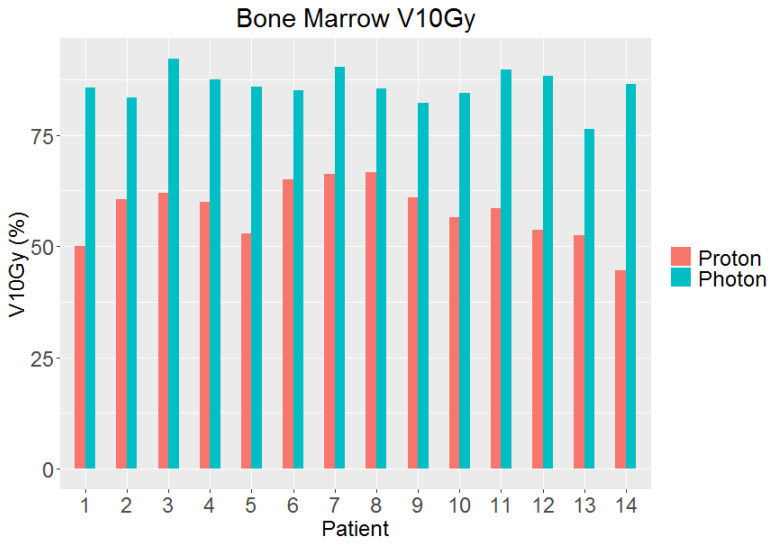
A comparison of the percentage of bone marrow that received at least 10 Gy on the proton and photon plans for each patient demonstrating a marked improvement for each patient with the proton plan compared to the photon plan.

The comparison between the DVH metrics from the proton and photon plans for the bladder, bone marrow, small bowel, and rectum is shown in the box plots in Figure 2. As bone marrow was the primary dose reduction endpoint, this structure was examined in more depth as shown in Figure 3, which shows the comparison of V10Gy for each patient under the photon and proton plans. This demonstrates a marked improvement for every patient, demonstrating the potential of IFSO to spare bone marrow.

The DVH metrics under the applied robustness criteria are summarized in Table 3. Pairwise *t*-tests between the photon and proton 5 mm robustness evaluations showed that all significant improvements in the proton plan remained, and additionally, the small bowel V40Gy was significantly improved. Similarly, pairwise *t*-tests between the photon 5 mm robustness and proton 5 mm/3% robustness evaluations showed that all significant improvements in the proton plan remained, except for the small bowel V35Gy and rectum mean dose.

**Figure 4:**
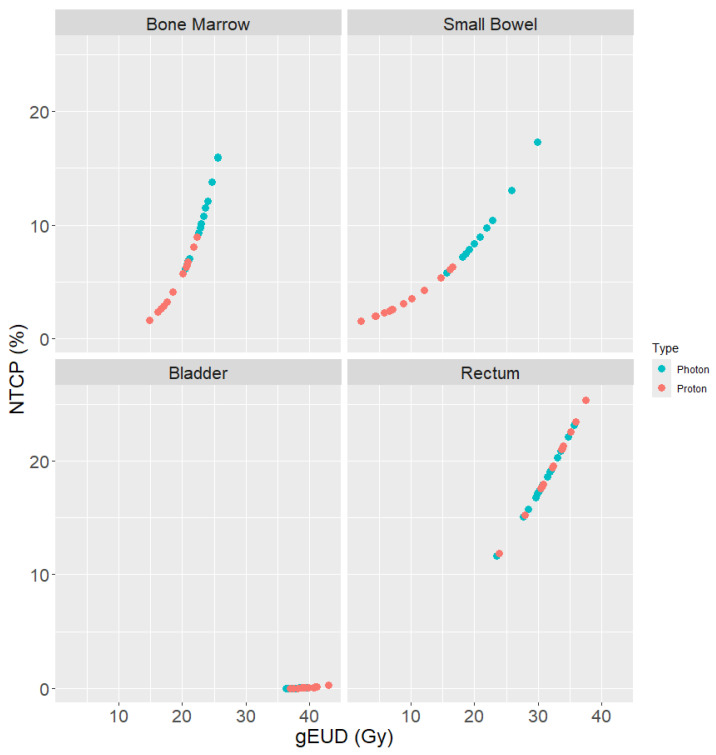
The NTCP for each patient’s proton and photon plan is plotted against the generalized equivalent uniform dose (gEUD) for the bone marrow, bladder, small bowel, and rectum. The bone marrow and small bowel are clearly separated between the two modalities, with photon having significantly higher toxicity and dose.

Figure 4 displays the generalized equivalent uniform dose vs. the NTCP for both the proton and photon plans for all OARs assessed. Based on the paired *t*-tests of the bladder NTCP evaluation, there was a significant increase (*p* = 0.004) in NTCP for the proton treatment plans. However, the mean NTCP percentage for both the proton and photon treatment plans was very low: 0.03% for photon plans and 0.07% for proton plans. Therefore, this is not a clinically significant increase. The bowel NTCP was significantly reduced (*p* < 0.001) from a mean of 9.39% for photon plans to 3.32% for proton plans. The rectum NTCP was not significantly different (*p* = 0.12) between the different modalities. The hematological toxicity was significantly reduced (*p* < 0.001) from a mean NTCP of 10.20% for photon plans to 4.91% for proton plans.

## 4. Discussion

The results of this study demonstrate that scanning beam proton therapy using the IFSO method yields substantial dosimetric benefits that may translate into meaningful clinical improvements for patients undergoing adjuvant radiotherapy for gynecologic cancers. Specifically, IFSO significantly reduces radiation exposure to critical OARs, especially the bone marrow and small bowel, two structures closely linked to acute and chronic treatment toxicities.

Clinically, bone marrow sparing is particularly significant because hematological toxicity is a common dose-limiting side effect of pelvic radiotherapy, especially when chemotherapy is administered concurrently or sequentially. This study showed large reductions in V10Gy and V20Gy to bone marrow with proton therapy, with associated decreases in the modeled probability of grade ≥ 3 hematological toxicities. These reductions are clinically meaningful, as hematologic toxicity can lead to treatment delays, increased infection risk, and the need for transfusions, ultimately compromising cancer treatment efficacy and patient quality of life.

The observed reduction in small bowel V20–V35Gy and associated NTCP modeling predict a lower risk of radiation-induced bowel urgency syndrome—an outcome that significantly affects daily functioning and long-term quality of life in gynecologic cancer survivors. These findings suggest that patients treated with IFSO-based proton therapy may experience fewer gastrointestinal symptoms during and after treatment.

Although the dosimetric advantage for the rectum was less pronounced, the reduction in mean dose still points to a potential decrease in low-grade rectal toxicity. Furthermore, although bladder sparing was not statistically significant and even showed a slight increase in NTCP modeling, the absolute probability of bladder toxicity remained very low for both modalities, implying limited clinical relevance.

The bladder NTCP was increased for proton plans, although not clinically meaningful, as both photon and proton NTCPs were below 0.1%. As Table 2 demonstrates, the mean bladder dose was reduced for proton planning, but V45Gy was increased, demonstrating a shift in the DVH. The proton plans limited the low-dose spread but had some higher doses near the bladder due to robustness considerations, which caused this shift in DVH and resultant higher NTCP calculation.

The robustness of IFSO plans under both setup and range uncertainty conditions underscores the practicality of this method for clinical implementation, provided appropriate resources and training are in place. While the planning process for IFSO is more labor-intensive and lacks skin sparing, these drawbacks are outweighed by the potential for improved clinical outcomes, especially in high-risk or medically vulnerable patient populations. A potential limitation of scanning beam proton therapy, particularly with multiple anterior fields, is higher skin dose, which was not directly evaluated in this study. Monitoring skin toxicity should be part of future prospective evaluations.

There are several limitations to this study. First, there is a limited number of patients included in this study. The sample size of 14 patients and the retrospective design limit generalizability. Secondly, although photon gynecological cancer patients are treated with a full bladder, this study utilized the proton patient dataset to allow direct dosimetric comparisons. Additionally, clinical outcomes such as toxicity or recurrence were not available, so we used dosimetric and NTCP modeling as surrogates. Future prospective studies are needed to validate these findings. Lastly, this is a single-institution study, and future studies should expand on this to compare how different centers’ implementation of IFSO and VMAT planning may impact these results.

As with all proton therapy techniques, there remains a risk of tumor underdosing due to range uncertainty. This is particularly important near air- or gas-filled organs like the bowel. Our robustness analysis incorporated ±3% range uncertainty to account for this, but clinical validation remains essential.

While the IFSO method offers substantial dosimetric and biological advantages, it is also more complex to implement. IFSO planning requires advanced treatment planning software capable of supporting multi-field robust optimization, increased computational time, and skilled personnel experienced in proton therapy contouring and optimization strategies. Additionally, the workflow involves intricate beam arrangement and contour cropping processes, which can significantly extend planning time compared to VMAT. In resource-limited or lower-volume centers, these demands may present a barrier to adoption. The need for institutional investment in training, planning systems, and clinical workflow support should be carefully considered when evaluating whether IFSO is a practical approach in such settings.

Importantly, this study adds to the growing body of evidence supporting the use of scanning proton therapy in gynecologic malignancies and is among the first to highlight the potential clinical relevance of split-target planning. As treatment techniques evolve, future prospective studies should assess whether the observed dosimetric benefits of IFSO translate into fewer acute and late toxicities, better treatment compliance, and improved patient-reported outcomes. This may be particularly relevant for patients receiving extended field radiation (e.g., para-aortic nodes) or concurrent chemotherapy, where normal tissue sparing is most needed.

## 5. Conclusions

Individual Field Simultaneous Optimization (IFSO) is a split-target method for spot-scanning proton planning that allows for robust plans with dosing that conforms to targets and spares OARs. IFSO significantly spares low-dose volumes in OARs compared to VMAT, particularly in the bone marrow and small bowel. Patient quality of life may be improved, and toxicities may be reduced with the dose sparing offered by IFSO.

## Figures and Tables

**Table 1:** Patient demographics.

Patient	Age (years)	FIGO ^†^	T	N	M	Volume of CTV (cm^3^)
1	61	IIIC1	T2	N1	M0	339.2
2	65	II	T2	N0	M0	327.1
3	78	IIIB	T3b	N0	M1	463.5
4	66	IIIC2	T1	N2	Mx	415.0
5 *	49		T4	N1	M0	599.3
6	69	IIIC1	T1c	N1	M0	315.3
7	83	IIIC1	T1b	N1b	M0	478.9
8	81	IIIC1	T1b	N1	M0	464.7
9	65	IIIC2	T1b	N2a	M0	487.3
10	83	IA	T1a	N0	M0	244.5
11	72	IA	T1a	Nx	M0	325.2
12	67	IA	T1a	N0	M0	316.2
13	76	IA	T1a	N0	M0	348.4
14	76	IIIA	T3a	N0	M0	376.8

* The patient had surgery at an outside institution, and the histological data were not sent. ^†^ FIGO 2009 staging harmonized with AJCC 8th edition.

**Table 3:** Dose volume histogram metrics for organs at risk under robustness criteria.

Organ	Metric	Photon 5 mm Robustness Mean (Range)	Proton 5 mm Robustness Mean (Range)	Proton 5 mm/3% Robustness Mean (Range)
Bladder	Mean	32.1 Gy (27.5–35.4 Gy)	31.8 Gy (25.3–40.5 Gy)	32.9 Gy (26.6–41.0 Gy)
	V20Gy	75.2% (57.1–88.3%)	73.5% (58.4–92.9%)	76.1% (63.9–96.8%)
	V45Gy	27.0% (17.5–42.7%)	29.8% (11.3–60.1%)	33.5% (13.2–63.0%)
Bone Marrow	V10Gy	87.0% (77.2–93.0%)	58.9% (46.1–67.9%)	59.1% (46.3–68.0%)
	V20Gy	59.3% (43.7–68.0%)	47.9% (36.0–59.4%)	48.3% (36.5–59.6%)
	V30Gy	28.1% (19.5–39.2%)	36.9% (30.1–45.1%)	37.8% (30.2–45.6%)
	V40Gy	13.2% (8.1–21.1%)	15.5% (9.0–22.5%)	16.8% (9.8–23.0%)
Femoral Head (left)	Mean	14.1 Gy (8.9–19.5 Gy)	12.3 Gy (5.8–18.8 Gy)	12.6 Gy (5.9–19.0 Gy)
Femoral Head (right)	Mean	14.0 Gy (9.4–20.1 Gy)	12.2 Gy (5.8–18.6 Gy)	12.4 Gy (5.9–18.8 Gy)
Rectum	Mean	25.4 Gy (16.8–30.4 Gy)	22.4 Gy (11.0–28.9 Gy)	24.1 Gy (11.7–33.7 Gy)
	V30Gy	39.8% (16.1–63.9%)	42.6% (17.3–59.4%)	46.7% (19.2–69.1%)
	V45Gy	15.7% (2.8–25.7%)	16.0% (5.1–31.1%)	21.0% (4.7–38.6%)
Small Bowel	V20Gy	59.6% (32.7–79.1%)	22.9% (6.7–56.4%)	26.9% (10.7–62.2%)
	V30Gy	28.7% (12.0–47.0%)	18.4% (4.3–48.8%)	22.1% (7.5–54.6%)
	V35Gy	20.8% (7.9–36.9%)	16.1% (3.4–43.7%)	19.6% (6.1–49.2%)
	V40Gy	15.3% (5.4–28.3%)	13.4% (2.6–37.6%)	16.6% (4.6–43.2%)
	V45Gy	9.7% (3.2–19.3%)	8.7% (0.9–28.0%)	10.5% (1.6–31.4%)

## Data Availability

The data presented in this study are available on request from the corresponding author. The data are not publicly available due to privacy or ethical restrictions.

## Abbreviations

The following abbreviations are used in this manuscript:

OAR	Organ at risk
IFSO	Individual Field Simultaneous Optimization
VMAT	Volumetric modulated arc therapy
NTCP	Normal tissue complication probability
sPBT	Scanning proton radiotherapy
CTV	Clinical target volume
OTV	Optimization target volume
STV	Scanning target volume
PTV	Planning target volume
DVH	Dose–volume histogram
gEUD	Generalized equivalent uniform dose

## References

[1] Torre L.A., Bray F., Siegel R.L., Ferlay J., Lortet-Tieulent J., Jemal A. (2015). Global Cancer Statistics, 2012. CA Cancer J. Clin..

[2] Mell L.K., Schomas D.A., Salama J.K., Devisetty K., Aydogan B., Miller R.C., Jani A.B., Kindler H.L., Mundt A.J., Roeske J.C. (2008). Association Between Bone Marrow Dosimetric Parameters and Acute Hematologic Toxicity in Anal Cancer Patients Treated With Concurrent Chemotherapy and Intensity-Modulated Radiotherapy. Int. J. Radiat. Oncol. Biol. Phys..

[3] Salama J.K., Mell L.K., Schomas D.A., Miller R.C., Devisetty K., Jani A.B., Mundt A.J., Roeske J.C., Liauw S.L., Chmura S.J. (2007). Concurrent Chemotherapy and Intensity-Modulated Radiation Therapy for Anal Canal Cancer Patients: A Multicenter Experience. J. Clin. Oncol..

[4] Milano M.T., Jani A.B., Farrey K.J., Rash C., Heimann R., Chmura S.J. (2005). Intensity-Modulated Radiation Therapy (IMRT) in the Treatment of Anal Cancer: Toxicity and Clinical Outcome. Int. J. Radiat. Oncol. Biol. Phys..

[5] van de Sande M.A.E., Creutzberg C.L., van de Water S., Sharfo A.W., Hoogeman M.S. (2016). Which Cervical and Endometrial Cancer Patients Will Benefit Most from Intensity-Modulated Proton Therapy?. Radiother. Oncol..

[6] De Boer P., Van de Schoot A.J.A.J., Westerveld H., Smit M., Buist M.R., Bel A., Rasch C.R.N., Stalpers L.J.A. (2017). PO-0833: Reducing Small Bowel Dose for Cervical Cancer Using IMPT and Target Tailoring in Treatment Planning. Radiother. Oncol..

[7] Anand A., Bues M., Rule W.G., Keole S.R., Beltran C.J., Yin J., Haddock M.G., Hallemeier C.L., Miller R.C., Ashman J.B. (2015). Scanning Proton Beam Therapy Reduces Normal Tissue Exposure in Pelvic Radiotherapy for Anal Cancer. Radiother. Oncol..

[8] Arians N., Lindel K., Krisam J., Oelmann-Avendano J.T., Meixner E., König L., Hoerner-Rieber J., Wark A., Forster T., Weykamp F. (2023). Treatment Tolerability and Toxicity of Postoperative Proton Beam Therapy for Gynecologic Malignancies: Results of the Prospective Phase 2 APROVE Trial. Int. J. Radiat. Oncol. Biol. Phys..

[9] Berlin E., Yegya-Raman N., Garver E., Li T., Lin L.L., Taunk N.K. (2023). Acute and Long-Term Toxicity of Whole Pelvis Proton Radiation Therapy for Definitive or Adjuvant Management of Gynecologic Cancers. Gynecol. Oncol..

[10] Taunk N. (2022). The Role of Proton Therapy in Gynecological Radiation Oncology. Int. J. Gynecol. Cancer.

[11] Anderson J.D., Voss M.M., Laughlin B.S., Garda A.E., Aziz K., Mullikin T.C., Haddock M.G., Petersen I.A., DeWees T.A., Vora S.A. (2023). Outcomes of Proton Beam Therapy Compared With Intensity-Modulated Radiation Therapy for Uterine Cancer. Int. J. Part. Ther..

[12] Song W.Y., Huh S.N., Liang Y., White G., Nichols R.C., Watkins W.T., Mundt A.J., Mell L.K. (2010). Dosimetric Comparison Study between Intensity Modulated Radiation Therapy and Three-Dimensional Conformal Proton Therapy for Pelvic Bone Marrow Sparing in the Treatment of Cervical Cancer. J. Appl. Clin. Med. Phys..

[13] Dinges E., Felderman N., McGuire S., Gross B., Bhatia S., Mott S., Buatti J., Wang D. (2015). Bone Marrow Sparing in Intensity Modulated Proton Therapy for Cervical Cancer: Efficacy and Robustness under Range and Setup Uncertainties. Radiother. Oncol..

[14] Wark A., Gupta A., Meixner E., König L., Hörner-Rieber J., Forster T., Lang K., Ellerbrock M., Herfarth K., Debus J. (2024). Bone Marrow Sparing by Intensity Modulated Proton Beam Therapy in Postoperative Irradiation of Gynecologic Malignancies. Technol. Cancer Res. Treat..

[15] Lin L.L., Kirk M., Scholey J., Taku N., Kiely J.B., White B., Both S. (2016). Initial Report of Pencil Beam Scanning Proton Therapy for Posthysterectomy Patients with Gynecologic Cancer. Int. J. Radiat. Oncol. Biol. Phys..

[16] Clivio A., Kluge A., Cozzi L., Köhler C., Neumann O., Vanetti E., Wlodarczyk W., Marnitz S. (2013). Intensity Modulated Proton Beam Radiation for Brachytherapy in Patients with Cervical Carcinoma. Int. J. Radiat. Oncol. Biol. Phys..

[17] Marnitz S., Wlodarczyk W., Neumann O., Koehler C., Weihrauch M., Budach V., Cozzi L. (2015). Which Technique for Radiation Is Most Beneficial for Patients with Locally Advanced Cervical Cancer? Intensity Modulated Proton Therapy versus Intensity Modulated Photon Treatment, Helical Tomotherapy and Volumetric Arc Therapy for Primary Radiation—An Intraindividual Comparison. Radiat. Oncol..

[18] Milby A.B., Both S., Ingram M., Lin L.L. (2012). Dosimetric Comparison of Combined Intensity-Modulated Radiotherapy (IMRT) and Proton Therapy versus IMRT Alone for Pelvic and Para-Aortic Radiotherapy in Gynecologic Malignancies. Int. J. Radiat. Oncol. Biol. Phys..

[19] Georg D., Georg P., Hillbrand M., Pötter R., Mock U. (2008). Assessment of Improved Organ at Risk Sparing for Advanced Cervix Carcinoma Utilizing Precision Radiotherapy Techniques. Strahlenther. Onkologie.

[20] Verma V., Simone C.B., Wahl A.O., Beriwal S., Mehta M.P. (2016). Proton Radiotherapy for Gynecologic Neoplasms. Acta Oncol..

[21] Hashimoto S., Shibamoto Y., Iwata H., Ogino H., Shibata H., Toshito T., Sugie C., Mizoe J.E. (2016). Whole-Pelvic Radiotherapy with Spot-Scanning Proton Beams for Uterine Cervical Cancer: A Planning Study. J. Radiat. Res..

[22] Anand A., Bues M., Gamez M.E., Stefan C., Patel S.H. (2019). Individual Field Simultaneous Optimization (IFSO) in Spot Scanning Proton Therapy of Head and Neck Cancers. Med. Dosim..

[23] Taku N., Dise J., Kenton O., Yin L., Teo B.K.K., Lin L.L. (2016). Quantification of Vaginal Motion Associated with Daily Endorectal Balloon Placement during Whole Pelvis Radiotherapy for Gynecologic Cancers. Radiother. Oncol..

[24] Michalski J.M., Gay H., Jackson A., Tucker S.L., Deasy J.O. (2010). Radiation Dose-Volume Effects in Radiation-Induced Rectal Injury. Int. J. Radiat. Oncol. Biol. Phys..

[25] Klopp A.H., Moughan J., Portelance L., Miller B.E., Salehpour M.R., Hildebrandt E., Nuanjing J., D’Souza D., Souhami L., Small W. (2013). Hematologic Toxicity in RTOG 0418: A Phase 2 Study of Postoperative IMRT for Gynecologic Cancer. Int. J. Radiat. Oncol. Biol. Phys..

[26] Kavanagh B.D., Pan C.C., Dawson L.A., Das S.K., Li X.A., Ten Haken R.K., Miften M. (2010). Radiation Dose-Volume Effects in the Stomach and Small Bowel. Int. J. Radiat. Oncol. Biol. Phys..

[27] Klopp A.H., Yeung A.R., Deshmukh S., Gil K.M., Wenzel L., Westin S.N., Gifford K., Gaffney D.K., Small W., Thompson S. (2018). Patient-Reported Toxicity during Pelvic Intensity-Modulated Radiation Therapy: NRG Oncology-RTOG 1203. J. Clin. Oncol..

[28] Feng Z., Tao C., Zhu J., Chen J., Yu G., Qin S., Yin Y., Li D. (2017). An Integrated Strategy of Biological and Physical Constraints in Biological Optimization for Cervical Carcinoma. Radiat. Oncol..

[29] Gulliford S.L., Partridge M., Sydes M.R., Webb S., Evans P.M., Dearnaley D.P. (2012). Parameters for the Lyman Kutcher Burman (LKB) Model of Normal Tissue Complication Probability (NTCP) for Specific Rectal Complications Observed in Clinical Practise. Radiother. Oncol..

[30] Yoshimura T., Kinoshita R., Onodera S., Toramatsu C., Suzuki R., Ito Y.M., Takao S., Matsuura T., Matsuzaki Y., Umegaki K. (2016). NTCP Modeling Analysis of Acute Hematologic Toxicity in Whole Pelvic Radiation Therapy for Gynecologic Malignancies—A Dosimetric Comparison of IMRT and Spot-Scanning Proton Therapy (SSPT). Phys. Medica.

[31] Alevronta E., Skokic V., Wilderäng U., Dunberger G., Sjöberg F., Bull C., Bergmark K., Jörnsten R., Steineck G. (2018). Dose-Response Relationships of the Sigmoid for Urgency Syndrome after Gynecological Radiotherapy. Acta Oncol..

